# Fatal tumoral hemorrhage from brain metastases of renal cell carcinoma after stereotactic radiotherapy and immune checkpoint inhibitor and vascular endothelial growth factor‐targeted therapy combinations

**DOI:** 10.1002/iju5.12708

**Published:** 2024-03-04

**Authors:** Kaoruko Iwasa, Shigeaki Nakazawa, Taigo Kato, Koji Hatano, Atsunari Kawashima, Shinichiro Fukuhara, Norio Nonomura

**Affiliations:** ^1^ Department of Urology Osaka University Graduate School of Medicine Suita Osaka Japan

**Keywords:** brain metastasis, fatal tumor hemorrhage, immune checkpoint inhibitor, renal cell carcinoma, stereotactic radiotherapy

## Abstract

**Introduction:**

Brain metastasis in renal cell carcinoma, which is reported in 10% of cases, leads to significant morbidity and mortality. Establishment of appropriate and safe treatment for brain metastasis renal cell carcinoma remains a pressing need.

**Case presentation:**

A 56‐year‐old female patient, presenting with anorexia, headache, and occipital swelling, was subsequently diagnosed with clear cell renal cell carcinoma with multiple metastases, including intracranial and epicranial tumors. The patient initially underwent stereotactic radiotherapy for metastatic brain tumors and then received combination therapy with pembrolizumab and lenvatinib. However, after 30 days of treatment, the patient experienced a sudden loss of consciousness due to massive multifocal intracranial hemorrhage, leading to her death the following day.

**Conclusion:**

Although fatal tumoral hemorrhage during combined stereotactic radiotherapy and immune checkpoint inhibitor/VEGF‐targeted therapy for patients with brain metastasis renal cell carcinoma is an extremely rare complication, it should always be considered a possibility.

**Keynote message**

A patient with brain metastases from renal cell carcinoma suffered a fatal cerebral hemorrhage shortly after the initiation of medical therapy. This should be recognized as a serious complication of this disorder.

**Abbreviations & Acronyms**
BM: brain metastasis
CT: computed tomography
ICH: intracranial hemorrhage
ICI: immune checkpoint inhibitor
OS: overall survival
RCC: renal cell carcinoma
SRT: stereotactic radiotherapy
TKI: tyrosine kinase inhibitor
VEGF: vascular endothelial growth factor

## Case presentation

**Fig. 1:**
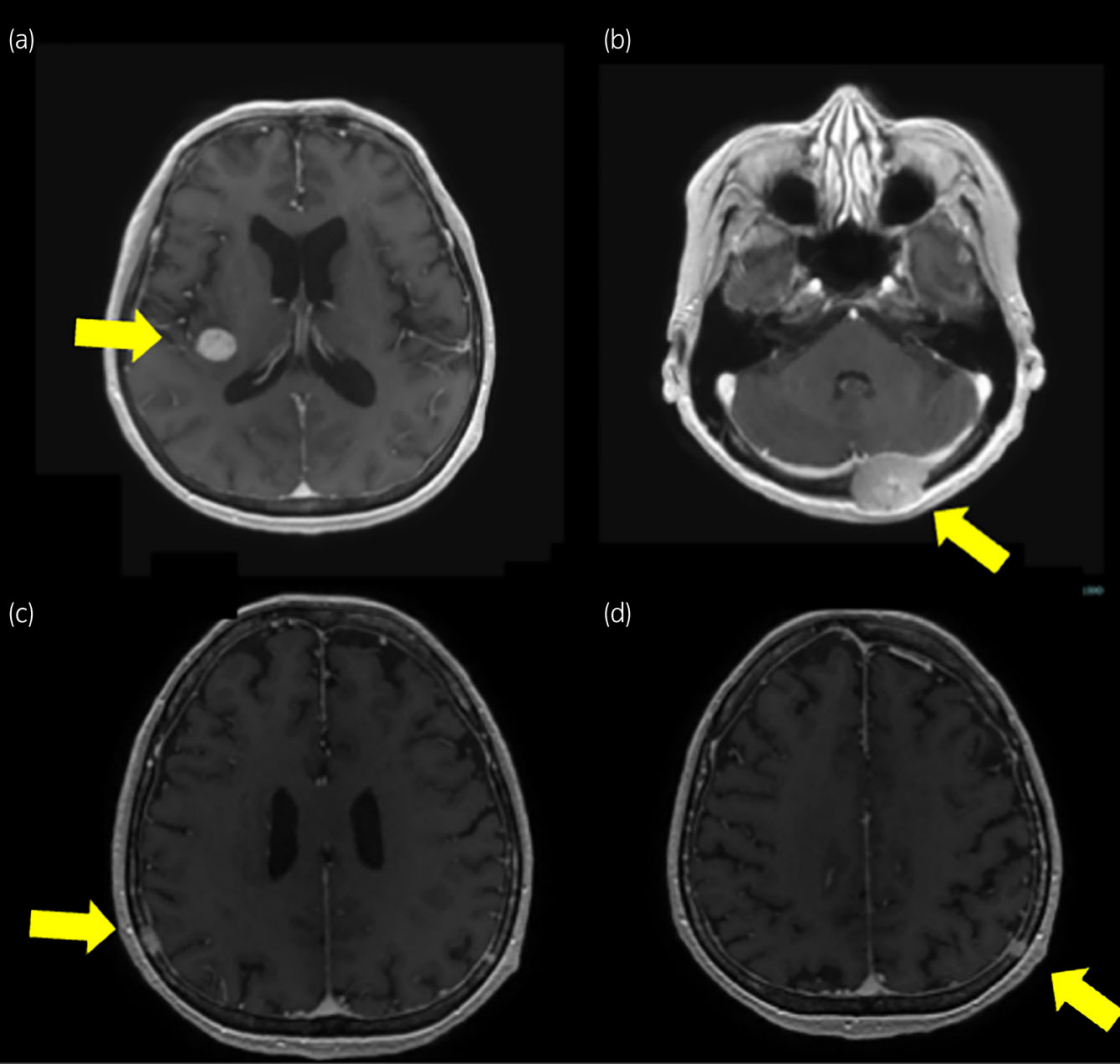
Magnetic resonance images. MR images of the head revealed an intracranial tumor in the right insula (a) and left occipital region with bone destruction (b). In addition, bilateral epidural tumors were observed (c, d). The yellow arrows indicate the positions of the tumors.

**Fig. 2:**
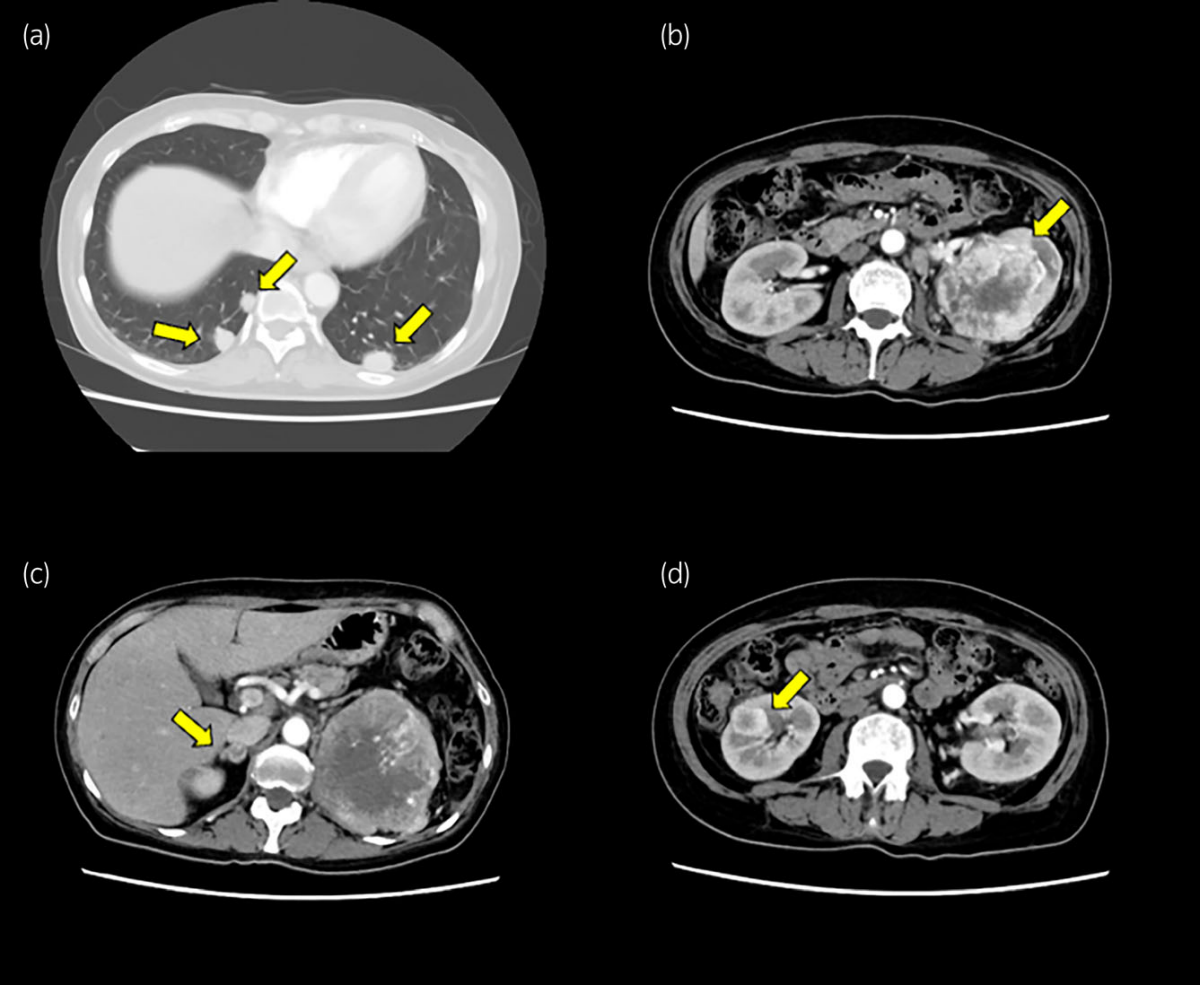
CT images. Contrast‐enhanced CT image showed multiple lung metastases (a), left renal tumor (b), right adrenal metastasis (c), and right renal metastasis (d) (yellow arrows).

A 56‐year‐old female patient presented with anorexia, a headache, and occipital swelling. The patient had lost 16 kg of body weight over the past 7 months due to anorexia. The neurosurgery department performed magnetic resonance imaging of the head, revealing multiple intracranial and epicranial tumors (Fig. 1). Following this, the patient was referred to our hospital for further examination. The patient had a history of left ovarian cyst and no oral medications. A thoracoabdominal CT scan showed left renal cancer with multiple metastases to the right kidney, right adrenal gland, and lung (Fig. 2). Blood tests indicated anemia (hemoglobin level: 8.7 mg/dL) and elevated corrected calcium (11.6 mg/dL), and C‐reactive protein (15.4 mg/dL) levels. Platelet count exceeded 500 000/μL, but no blood coagulation abnormalities were observed. The pathology results of the CT‐guided needle biopsy of the left renal tumor confirmed clear cell RCC, staged as cT3aN1M1, and the patient's risk status according to the International Metastatic RCC Database Consortium was poor.

**Fig. 3:**
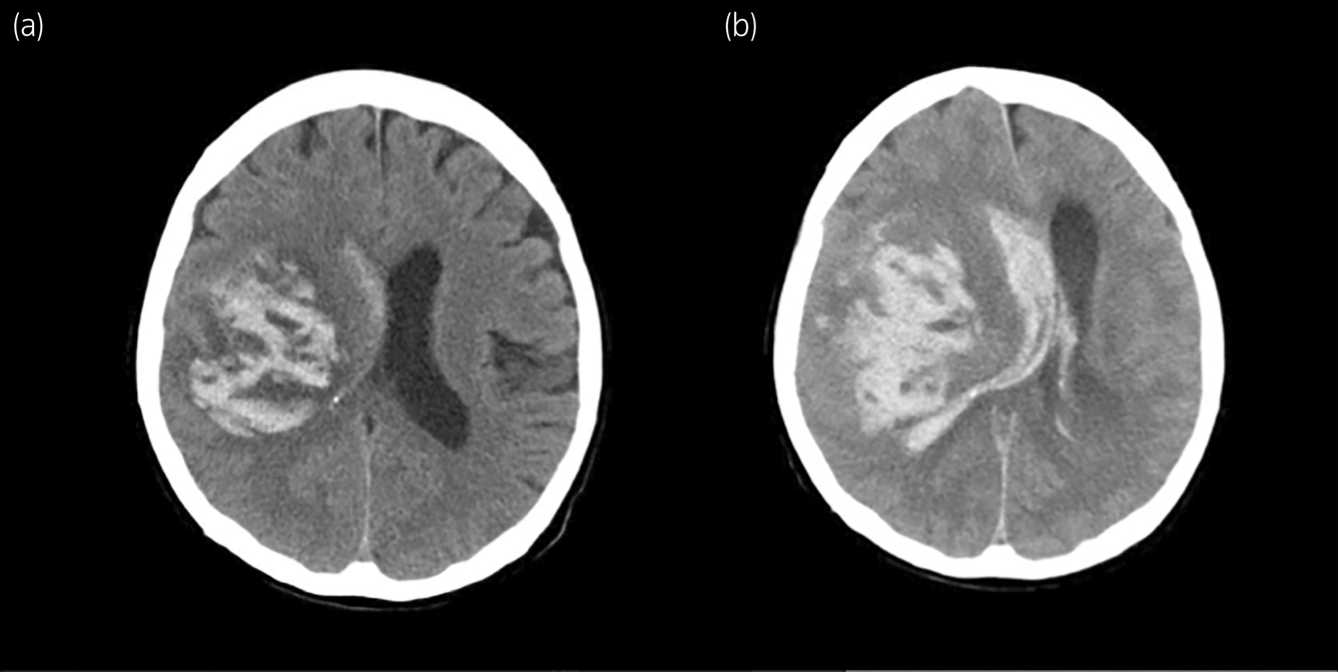
CT images of the cerebral hemorrhage. CT scans of the head revealed a cerebral hemorrhage appearing to originate from a metastatic lesion in the right insular cortex. The left image was taken immediately after the hemorrhage (a), and the right image was taken 6 h after the hemorrhage (b).

**Fig. 4:**
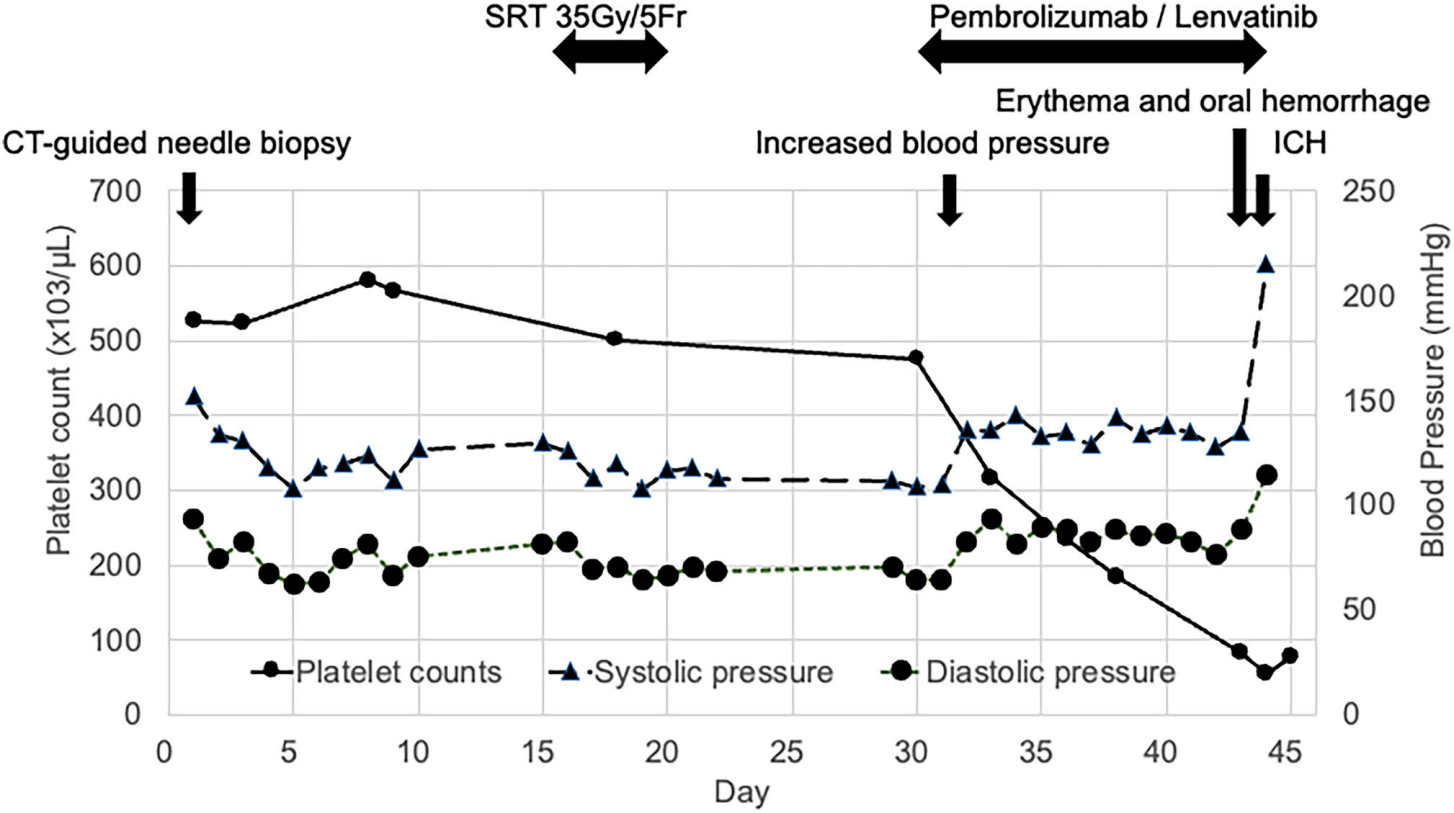
Clinical course. SRT, stereotactic radiotherapy; ICH, intracranial hemorrhage.

Initially, SRT was performed for the metastatic brain tumors, and a prescription dose of 35 Gy was selected for all metastatic brain lesions. After 17 days, the patient began combination therapy with pembrolizumab (200 mg) and lenvatinib (20 mg). The next day, mildly elevated blood pressure (Common Terminology Criteria for Adverse Events v5.0 grade 1) was observed. On day 29, erythema on the face and trunk (grade 1) and oral hemorrhage (grade 1) were observed, which were considered side effects of lenvatinib, and the platelet count decreased to 87 000/μL (grade 1). On day 30, the patient suddenly lost consciousness, with a massive increase in blood pressure (grade 4). Emergency CT scans revealed a massive multifocal ICH, likely originating from the irradiated tumors, that caused a midline shift of the brain (Fig. 3a). Despite intensive treatment to reduce intracranial pressure, the cerebral hemorrhage worsened, and the patient died the following day (Figs 3b,4).

## Discussion

Approximately 10% of patients with RCC develop BM, significantly impacting morbidity and mortality.^1^ Patients with untreated BM have a poor prognosis, with short progression‐free survival and OS. A retrospective review reported a median OS of 10.3 months (range, 7.0–17.9 months) and a 1‐year OS probability of 48% (95% CI, 37–62).^2^

In the current era, new therapeutic options for RCC are emerging, counting TKIs, mostly VEGF receptor inhibitors, and ICIs.^3^ The KEYNOTE‐581/CLEAR study highlighted the efficacy of pembrolizumab and lenvatinib, positioning it as a primary treatment for advanced clear cell RCC.^4^ These new therapeutic approaches have extended the median survival of BM patients to approximately 14 months, although this is still less than those without BM.^5^, ^6^, ^7^ At our institution, the combination therapy of pembrolizumab and lenvatinib is set as the first‐line treatment for metastatic RCC.

According to the latest evidence, BM RCC is primarily managed with neurosurgery and/or radiotherapy, with additive therapeutic benefits from systemic therapy, such as ICIs or TKIs.^3^, ^8^ While RCC has traditionally been considered radioresistant, recent findings suggest fractionated high‐dose radiotherapy could be beneficial. SRT has been recognized as a safe and effective treatment for small, solitary, and multiple metastatic brain tumors, offering high local control.^9^, ^10^ On the other hand, ICH after SRT has been reported in several cases.

ICH is more frequent in metastatic tumors than primary ones, and its incidence varies with the type of malignancy. Pathological and radiological studies suggest that ICH in intracranial neoplasms occurs in 1.4–10% of cases, averaging around 2–3%.^11^, ^12^ RCC metastases, in particular, are prone to spontaneous bleeding,^13^ with a reported 12% rate of delayed hemorrhage in a large patient series.^14^ A comprehensive retrospective study encompassing various cancer types revealed ICH rates of 0.53% per patient, 0.33% per intervention, and 0.08% per lesion.^15^

ICH from BM RCC can occur due to various factors. Firstly, RCC often have a rich blood supply and can lead to vascular vulnerability. Secondly, the use of TKIs targeting the VEGFR has been linked to a high frequency of fatal intracerebral hemorrhage.^16^ Thirdly, high‐dose radiation may have acute effects on abnormal tumor endothelial cells, potentially increasing the risk of intratumoral hemorrhage following radiotherapy.^17^ In addition, it is suggested that the thrombocytopenia caused by lenvatinib may have decreased coagulability.

A retrospective study investigating the effects of targeted therapies as add‐ons to radiation therapy has shown improvements in both local disease control and OS without increasing neurological mortality.^18^ However, no conclusions have been reached regarding which drugs should be used as add‐ons. Hirsch *et al*.^19^ evaluated the effectiveness and side effects of cabozantinib in conjunction with brain‐focused therapy for BM RCC. The study reported encouraging results, including an extended median OS of 16 months (95% CI, 12.0–21.9 months). In this study, cabozantinib was well‐tolerated, and no treatment‐related deaths were observed. The CheckMate 920 trial, a multi‐cohort phase 3b/4 study, assessed the safety and efficacy of nivolumab and ipilimumab in a group including patients with non‐clear cell RCC and BM.^20^ The trial observed no severe immune‐mediated adverse events, and the median OS was 21.2 months (95% CI, 16.6 months to not estimable), with both treatments being well‐tolerated and showing relatively positive outcomes. It is essential to establish an optimal treatment protocol in the future, as more case data accumulates.

## Conclusion

The presented case highlights the challenges in managing BM RCC. SRT has shown promising results with high local control in the treatment of patients with small solitary and multiple metastatic brain tumors. However, the incidence of ICH in metastatic tumors, especially RCC, should be considered when choosing the appropriate treatment modality. Further research is needed to evaluate the effects of ICIs and other targeted therapies on metastatic brain lesions. Moreover, novel treatment strategies should be developed to address the unmet clinical need for management of BM RCC.

## Author contributions

Shigeaki Nakazawa: Writing – original draft; writing – review and editing. Kaoruko Iwasa: Writing – original draft; writing – review and editing. Taigo Kato: Writing – review and editing. Koji Hatano: Writing – review and editing. Atsunari Kawashima: Writing – review and editing. Shinichiro Fukuhara: Writing – review and editing. Norio Nonomura: Writing – review and editing.

## Conflict of interest

The authors declare no conflict of interest.

## Approval of the research protocol by an Institutional Reviewer Board

Not applicable.

## Informed consent

Written informed consent was obtained from the patient for publication of this case report and the accompanying images.

## Registry and the Registration No. of the study/trial

Not applicable.
